# Clinical effects of dupilumab: A novel treatment for Kimura disease

**DOI:** 10.1002/iid3.1084

**Published:** 2023-11-08

**Authors:** Bing‐Shiau Shang, Chang‐Hung Hsiao, Teng‐Fu Tsao, Yuan‐Ya Liao, Wea‐Lung Lin, Wen‐I Lee, Ko‐Huang Lue

**Affiliations:** ^1^ Department of Pediatrics Chung Shan Medical University Hospital Taichung City Taiwan; ^2^ Department of Medicine Chung Shan Medical University Taichung City Taiwan; ^3^ Institute of Medicine Chung Shan Medical University Hospital Taichung City Taiwan; ^4^ Department of Pediatrics Cheng Ching Hospital Taichung City Taiwan; ^5^ Department of Medical Imaging Chung Shan Medical University Hospital Taichung City Taiwan; ^6^ Department of Surgery Chung Shan Medical University Hospital Taichung City Taiwan; ^7^ Department of Pathology Chung Shan Medical University Hospital Taichung City Taiwan; ^8^ Department of Pathology Chung Shan Medical University Taichung City Taiwan; ^9^ Department of Pediatrics, Division of Allergy, Asthma, Immunology and Rheumatology Chang Gung Memorial Hospital and Chang Gung University College of Medicine Taoyuan Taiwan; ^10^ Primary Immunodeficiency Care and Research Institute Chang Gung University College of Medicine and Chang Gung Memorial Hospital Taoyuan Taiwan; ^11^ Department of Biological Science & Technology National Yang Ming Chiao Tung University Taipei Taiwan

**Keywords:** dupilumab, Kimura disease, nephrotic syndrome, pediatric

## Abstract

**Background:**

Kimura disease (KD) is a rare chronic inflammatory disorder involving the Th2 pathway. Although medical treatment with steroids or other immunosuppressants is available, they may cause developmental issues in the pediatric population. Surgical intervention has also been suggested; however, it is associated with high recurrence rates.

**Case Presentation:**

A 14‐year‐old boy presented with left retroauricular lymph node enlargement at the age of 5 years. At the age of 7 years, he was diagnosed with nephrotic syndrome which subsided after steroid treatment for approximately 6 years. The retroauricular lymph node was surgically excised, and KD was confirmed. However, recurrent enlargement of the left retroauricular and neck lymph nodes occurred after 2 years. Persistently high IgE levels and fluctuating eosinophil counts were observed following steroid treatment. Dupilumab was prescribed because of the difficulty in tapering the steroid dosage. A loading dose of 600 mg was administered, followed by a maintenance dose of 300 mg every 2 weeks. The IgE level decreased after 3 months, and a low eosinophil count was maintained after steroid discontinuation. Follow‐up computed tomography revealed a decrease in the size of the lymph nodes with no side effects such as conjunctivitis.

**Conclusion:**

Traditional treatments have raised developmental concerns in the pediatric population and are associated with high recurrence rates. Dupilumab targets the Th2 pathway and provides effective results, with few adverse effects. Dupilumab may be a therapeutic option for KD and other diseases involving the Th2 pathway.

## Graphical abstract

Kumura disease is rare and mainly treated with corticosteroids, immunosuppressant or surgery. These treatment remained high recurrent rate and raised developmental concerns in children. Dupilumab presented as a novel treatment with less side effects and developmental problems

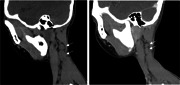

## 1 BACKGROUND

Kimura disease (KD) is rare and primarily affects young males in Asia, presenting as subcutaneous masses or lymphadenopathies in the head and neck regions. Renal complications are also common, with an incidence of 10–60%, of which 59–78% patients develop nephrotic syndrome.^1^ Its pathogenesis is still largely unknown, but involvement of the Th2 pathway has been suggested.^2^ Our patient was a 14‐year‐old boy with recurrent left retroauricular and neck lymphadenopathy along with elevated serum eosinophil count and IgE levels. The patient was treated with dupilumab based on the possible pathogenic involvement of the Th2 pathway.

## 2 CASE PRESENTATION

A 14‐year‐old boy was born at the gestational age of 40 weeks via a normal spontaneous delivery to a gravida 1, para 1. He was brought to our hospital at 5 years of age because of decreased height after 1 year of age and left retroauricular lymph node enlargement in the previous 3 months. Laboratory examination revealed no significant abnormalities, and the lymph nodes were managed with further observation. Two years later, he was diagnosed with nephrotic syndrome with an IgE level of >2000 kU/L, and an eosinophil count that fluctuated (0–29.8%) with steroid treatment. He had received steroid treatment for approximately 6 years. The patient had been proteinuria‐free for more than 1 year since the age of 13 years.

**Figure 1:**
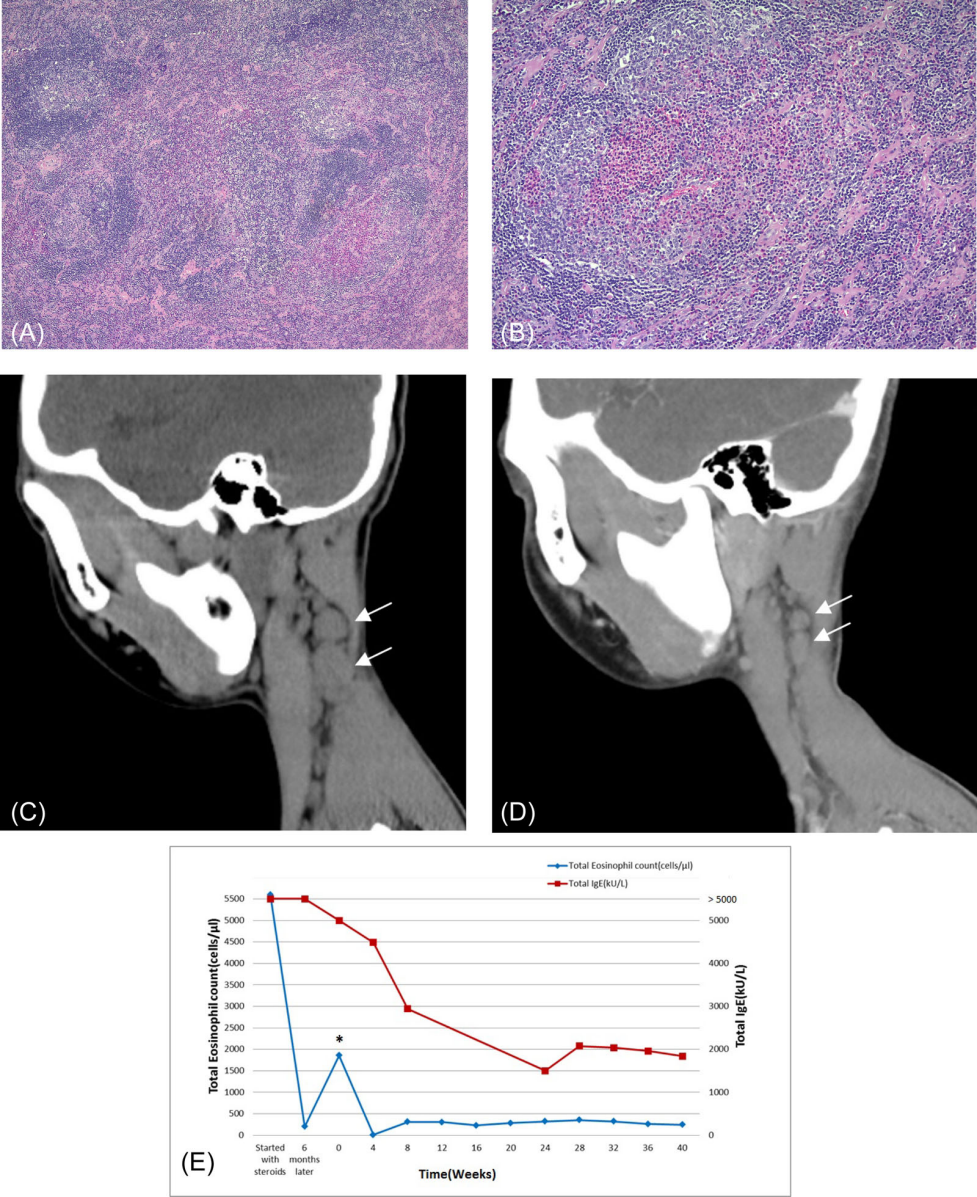
A. Lymphoid follicle hyperplasia with enlarged germinal center and increased eosinophils in the interfollicular area are prominent. Vascular proliferation and a distinct mantle zone are present. (H&E stain, ×20). Figure B. Infiltration of lymphocytes, plasma cells, many interfollicular eosinophils and hyalinized small blood vessels are noted. (H&E stain, ×100). Figure C. Neck computed tomography (CT) without contrast showing left neck lymph nodes measuring 1.1 × 1.3 and 2.4 × 1.6 cm. Figure D. Neck CT with contrast showing left neck lymph nodes measuring 0.68 × 0.8 and 1.2 × 0.7 cm 3 months after dupilumab treatment (white arrows). Figure E. Total eosinophil count and total IgE level. Dupilumab was added at Week 0 and steroids were discontinued at Week 8. *Steroids were tapered below 15 mg/day for 1 month and then dupilumab was added.

At the age of 12 years, he visited our pediatric surgery clinic for the evaluation of a 3 × 3 cm left retroauricular subcutaneous mass, which had been present for years but had gradually increased in size. His white blood cell (WBC) count was 10510/μL with 21% eosinophils. Surgical excision revealed a 2.5 × 1.7 × 1.0 cm lymph node, and KD was diagnosed based on the pathology (Figures 1A,B).

Two years postoperatively, the patient presented with a 2 × 1‐cm lymph node located inferior to the left retroauricular area, which was noted 1 month prior. Laboratory examination results revealed the following: WBC: 10330/μL, eosinophil: 23.5%, IgE level of >5000 kU/L, and an antinuclear antibody test at 1:160 of the positive homogeneous type. Additionally, C4 (10.1 mg/dL) was low, C3 (97.2 mg/dL) was within the normal range, and the anti‐dsDNA, anti‐Sm, or anti‐SSA/SSB antibodies were not elevated. HyperIgE and hypereosinophil syndrome were also considered and excluded. Relapsed KD was suspected; however, the patient's parents refused further biopsy.

Further elevation of eosinophils was noted (up to 39.3%), total eosinophil count was 5600/μL, with an IgE level of >5000 kU/L. Multiple palpable lymph nodes ranging from 1 to 2.5 cm in size were observed in the right side of the neck. Neck computed tomography (CT) without contrast revealed bilateral neck lymph nodes with two predominant nodes in the left neck were 1.1 × 1.3 and 2.4 × 1.6 cm in size (Figure 1C). Prednisolone was prescribed as his parents refused irradiation or surgical therapy. After 5 weeks of treatment, the size of the two lymph nodes decreased to 1.0 × 0.8 and 1 × 0.7 cm, respectively, while the other nodes were non‐palpable, and the eosinophil count dropped to 0.6%. Prednisolone was gradually tapered over 5 months; however, the eosinophil count increased back to 23.7% (1860/μL) and the two left lymph nodes showed mild enlargement with sizes of 1.4 × 1 and 2 × 1 cm when the dosage was below 15 mg/day. In addition, the IgE level remained at >5000 kU/L after 6 months.

Based on the possible pathogenesis of KD and the difficulty in tapering corticosteroid use, we added dupilumab for further treatment with his parents' agreement. We prescribed a 600 mg loading dose followed by 300 mg dupilumab every 2 weeks. Corticosteroids were discontinued 2 months after dupilumab administration. Three months after the addition of dupilumab, a neck CT revealed that the left lymph node had decreased in size (Figure 1D). The IgE level dropped to 2940 kU/L approximately 4 months later and was approximately 1500–2000 kU/L. The eosinophil percentage remained within 2.8–4.5% (310–357/μL) after corticosteroid discontinuation (Figure 1E). Currently, the patient is receiving dupilumab without significant side effects. We are closely monitoring the safety and efficacy of dupilumab therapy.

## 3 DISCUSSION AND CONCLUSION

KD may show renal involvement, with a frequency varying from 15–18% to 10–60%; however, there are only a few pediatric cases. This disease has various pathological manifestations, including minimal change disease, focal segmental glomerulosclerosis, mesangioproliferative glomerulonephritis, membranous nephropathy,^3^ and IgA nephropathy.^4^ Our patient presented with nephrotic syndrome after developing a post‐auricular mass but before the histological confirmation of KD. The differential diagnosess of kidney involvement with eosinophilia or elevated serum IgE levels was idiopathic nephrotic syndrome such as minimal‐change disease, IgA nephropathy, mesangioproliferative glomerulonephritis, membranous nephropathy, acute interstitial nephritis, DRESS syndrome, eosinophilic granulomatosis with polyangiitis, or chronic kidney disease. Renal biopsy was not performed because minimal‐change disease is the most common type of nephrotic syndrome in children with Th2 pathway involvement, often with elevated serum IgE level and some increased eosinophil count^5^, ^6^, ^7^; and our patient demonstrated an excellent response to steroid treatment, despite an episode of relapse.

However, the pathogenesis underlying the relationship between KD and nephrotic syndrome remains unclear. However, elevated IgE levels and eosinophilia have been observed in nephrotic syndrome. Therefore, the Th2 pathway may be involved in both diseases. Katagiri et al. reported higher expression of interleukin (IL)‐4, IL‐5, and IL‐13 mRNA in peripheral blood mononuclear cells from patients with KD compared to healthy controls.^2^ Similarly, Kimura et al. found that the number of EG2 + /IL‐4 + /IL‐5 + /Eotaxin + /RANTES + /CCR3+ cells was higher in KD.^8^ Elevated IL‐5 levels induce differentiation, proliferation, and activation of eosinophils. IL‐4 and IL‐13 induce an isotype switch in B‐cells to produce IgE. Hsiang et al. reported the development of KD in patients after allograft failure due to chronic rejection, and the eosinophil percentages ranging from 10% to 51%.^9^ Chronic rejection and preferential activation of CD4 + Th2 cells lead to activation of the Th2 pathway, which may explain this association.

KD lesions may precede, coincide with, or develop after renal involvement. Laboratory analyses often reveal elevated IgE levels and eosinophilia. The treatment options include surgical resection, corticosteroids, cyclosporine, and irradiation. However, the recurrence rate after surgical resection is 25%.^10^ Successful treatment with cyclosporine as a substitute for long‐term corticosteroids has been reported.^11^ Nonaka et al. showed the efficacy of treatment with an anti‐IgE agent (omalizumab) but found no change in serum IgE level and a high eosinophil count after 16 weeks.^12^ In our patient, we attempted to taper the steroid dose; however, treatment failure occurred when the dose reached <15 mg. Therefore, we considered dupilumab as a new therapy based on the involvement of the Th2 pathway in KD.

Dupilumab is a humanized IgG4 monoclonal antibody targeting the IL‐4 receptor alpha chain (IL‐4Rα), which inhibits both the IL‐4 and IL‐13 pathways.^13^ Dupilumab has been proven safe and effective in patients with moderate‐to‐severe atopic dermatitis and has improved lung function in patients with uncontrolled moderate‐to‐severe asthma with higher initial eosinophil or FeNO levels. In adults with chronic rhinosinusitis and nasal polyps, dupilumab reduces polyp size, sinus opacification, and symptom severity.^14^ Dupilumab can modulate and decrease the chemokines involved in type 2 inflammation, and the inhibition of IL‐4 and IL‐13 by dupilumab also decreases the production of IgE. Although a transient elevation of eosinophil count was observed approximately 4–16 weeks after the initial dose, it returned to baseline values or lower after 24 weeks in most patients, potentially due to an inhibition of eosinophil recruitment from the blood to the inflamed tissue, however, this is rare with eosinophilia‐related clinical symptoms.^15^, ^16^ There have been some reports on the effective use of dupilumab for the treatment of KD, with or without surgical excision. However, no pediatric reports or treatments for renal involvement have been published to date (Table 1).^17^, ^18^, ^19^, ^20^, ^21^ Our patient presented with persistently high levels of serum IgE despite corticosteroid treatment and rebounded eosinophilia with mild enlargement of the lymph nodes after the corticosteroids were tapered. Thus, we chose dupilumab as a treatment option because it decreases both eosinophil count and serum IgE levels, making it more effective than omalizumab. The side effects include local reactions at the injection sites, conjunctivitis, and hypersensitivity/anaphylaxis. However, most local reactions were mild, and the conjunctivitis was satisfactorily treated.

**Table 1:** Case reports of Kimura diseases treated by dupilumab.

	Age (year)/Sex	Clinical presentation	Treatment and results	Follow‐up	Recurrence
Bellinato et al.^17^	59/Male	Right zygomatic subcutaneous nodule s/p corticosteroids, tetracycline treatment.	Surgical excision + dupilumab 300 mg every 2 weeks. Improvement of nodule after 2 months.	6 months	No
Huang et al.^18^	36/Male	Right medial thigh mass for 2 years, no treatment was received.	Surgical excision + dupilumab 600 mg loading dose then 300 mg every 2 weeks. Serum IgE decreased from 8050 IU/mL to 2140 IU/mL after 16 months.	1 year	No
Suga et al.^19^	65/Male	Right subaural mass s/p excision then relapse on neck and back 22 years later.	Dupilumab 600 mg loading dose then 300 mg every 2 weeks. Eosinophil count and serum IgE decreased after 9 months.	15 months	No
Yang et al.^20^	57/Male	Bilateral anterior and posterior auricular mass, s/p corticosteroid for 1 year and recurrence after tapering. Then treatment with omalizumab for 19 cycles without improvement of mass and both elevated eosinophil and high serum IgE levels.	Dupilumab 600 mg loading dose them 300 mg every 2 weeks. Improvement of mass size and eosinophil returned to normal after 4 months.	4 months	No
Teraki et al.^21^	57/Male	Left arm subcutaneous nodule.	Dupilumab 600 mg loading dose then 300 mg every 2 weeks. Improvement of mass size, eosinophil count and serum IgE level after 4 months.	10 months	No

Dupilumab has been approved for the treatment of allergic diseases involving the Th2 pathway and its safety has been documented. In this report, we describe the use of dupilumab for KD, with no significant side effects. Dupilumab may be considered a treatment option based on the involvement of the Th2 pathway in KD.

## AUTHOR CONTRIBUTIONS

**Bing‐Shiau Shang**: Data curation; writing—original draft. **Chang‐Hung Hsiao**: Data curation; writing—original draft. **Teng‐Fu Tsao**: Data curation; visualization. **Yuan‐Ya Liao**: Writing—review and editing. **Wea‐Lung Lin**: Data curation. **Wen‐I Lee**: Formal analysis; writing—review and editing.

## CONFLICT OF INTEREST STATEMENT

The authors declare no conflict of interest.

## ETHICS STATEMENT

Informed consent for the study and its publication was obtained from the patient's parents.

## Data Availability

Data sharing is not applicable to this article as no new data were created or analyzed in this study.
